# Dental Attendances to General Medical Practitioners in Wales: A 44 Year-Analysis

**DOI:** 10.1177/00220345211044108

**Published:** 2021-09-28

**Authors:** C.C. Currie, S.J. Stone, P. Brocklehurst, G. Slade, J. Durham, M.S. Pearce

**Affiliations:** 1 School of Dental Sciences, Newcastle University, Newcastle upon Tyne, UK; 2 Newcastle upon Tyne NHS Foundation Trust, Newcastle upon Tyne, UK; 3 School of Health Sciences, Bangor University, Bangor, UK; 4 Division of Pediatric and Public Health, UNC Adams School of Dentistry, University of North Carolina, Chapel Hill, NC, USA; 5 Population Health Sciences Institute, Newcastle University, Newcastle upon Tyne, UK

**Keywords:** dental care, primary health care, toothache, antibacterial agents, epidemiology, public health

## Abstract

One-third of the UK population is composed of problem-oriented dental attenders, seeking dental care only when they have acute dental pain or problems. Patients seek urgent dental care from a range of health care professionals, including general medical practitioners. This study aimed to identify trends in dental attendance at Welsh medical practices over a 44-y period, specifically in relation to dental policy change and factors associated with repeat attendance. A retrospective observational study was completed via the nationwide Secure Anonymised Information Linkage (SAIL) Databank of visits to general medical practice in Wales. Read codes associated with dental diagnoses were extracted for patients attending their general medical practitioner between 1974 and 2017. Data were analyzed with descriptive statistics and univariate and multivariable logistic regression. Over the 44-y period, there were 439,361 dental Read codes, accounting for 288,147 patient attendances. The overall attendance rate was 2.60 attendances per 1,000 patient-years (95% CI, 2.59 to 2.61). The attendance rate was negligible through 1987 but increased sharply to 5.0 per 1,000 patient-years in 2006 (95% CI, 4.94 to 5.09) before almost halving to 2.6 per 1,000 in 2017 (95% CI, 2.53 to 2.63) to a pattern that coincided with changes to National Health Service policies. Overall 26,312 patients were repeat attenders and were associated with living in an area classified as urban and deprived (odds ratio [OR], 1.22; 95% CI, 1.19 to 1.25; *P* < 0.0001) or rural (OR, 0.84; 95% CI, 0.83 to 0.85; *P* < 0.0001). Repeat attendance was associated with greater odds of having received an antibiotic prescription (OR, 2.53; 95% CI, 2.50 to 2.56; *P* < 0.0001) but lower odds of having been referred to another service (OR, 0.75; 95% CI, 0.70 to 0.81; *P* < 0.0001). Welsh patients’ reliance on medical care for dental problems was influenced by social deprivation and health policy. This indicates that future interventions to discourage dental attendance at medical practitioners should be targeted at those in the most deprived urban areas or rural areas. In addition, health policy may influence attendance rates positively and negatively and should be considered in the future when decisions related to policy change are made.

## Introduction

One-third of UK adults do not seek routine dental care, instead attending only with acute dental pain or dental problems (Morris et al. 2011; Steele et al. 2011). This attendance pattern is not exclusive to the United Kingdom, with global estimates suggesting that just over half of dental patients see a dentist for preventive or regular dental care (Reda et al. 2018). Patients with acute dental pain present to a range of health care providers in addition to dentists (general dental practitioners [GDPs]): hospital emergency departments (Currie et al. 2017), pharmacists (Cohen, Bonito, et al. 2009), and general medical practitioners (GMPs; Anderson et al. 1999; Cope et al. 2016). Attendance at practitioners other than GDPs may not be the most appropriate place for patients to seek dental care, with operative dental treatment not being available resulting in potentially inappropriate antibiotic prescriptions, direct, indirect, and opportunity costs, as well as additional burden on health care services (Cope, Wood, et al. 2018).

Patients may prefer to seek care dental care from a GMP rather than a dentist for various reasons: interpretation of symptoms, perceptions of GMP scope of practice, comparative ease of navigating medical and dental systems, previous dental experiences, and willingness and ability to pay for treatment (Bell et al. 2008; Cope et al. 2015; Cope, Butt, and Chestnutt 2018; Cope, Wood, et al. 2018). GMP guidance and legislation within the United Kingdom highlights that presenting to GMPs with dental complaints is inappropriate and patients should be referred to a dentist or local emergency service (General Practitioners Committee 2007). This means that an initial GMP attendance for a dental problem may be justified if the patient was unaware of this; however, during this appointment, a referral should be given to the patient, meaning that any subsequent attendances for dental problems would be inappropriate.

The rate of dental attendances (calculated as an incidence rate) at UK GMPs is 6.06 consultations per 1,000 patient-years (Cope et al. 2016). While there are no recent comparable data for Wales, in 1996 the rate was greater at 6.90 per 1,000 patient-years (Anderson et al. 1999). The rate of dental visits at GMPs varies by practice, with attendances as often as once a week to once every few months (Cope et al. 2015).

**Figure 1.**
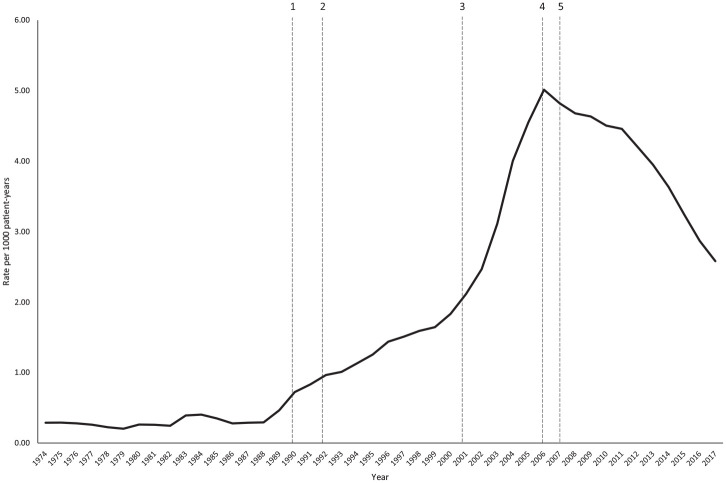
Rates of dental attendances over the period studied with key policy change dates labeled. (1) Introduction of capitation payments in NHS dentistry. GDPs were remunerated for the number of patients registered at their practices, resulting in an increase in patients registered as seeing a GDP. (2) Clawback of GDP fees due to overperformance from the changes in 1990. This led to a dispute between GDPs and the Department of Health initiating access issues for NHS dentistry. (3) Introduction of free dental check-ups to those <25 y and >60 y in Wales. (4) Introduction of a new NHS dental contract with loss of patient registration and capitation payments and introduction of a new payment model for NHS dentists, resulting in more dentists moving from the NHS to private dentistry. Change in provision of dental care in Wales, with responsibility for providing care moved to local health boards from individual practices. (5) Introduction of NHS Direct Wales, a free-to-use national telephone service for advice and access to nonemergency medical and dental services. GDP, general dental practitioner; NHS, National Health Service.

Dental attendances at GMPs could vary in relation to policy change. Within Wales, there have been several key policy changes that may affect patients’ decisions on where to seek dental care (Fig. 1):

- Introduction of capitation payments in NHS dentistry in 1990, where GDPs were remunerated for the number of patients registered at their practices
- Clawback of GDP fees in 1992 due to overperformance from the changes in 1990
- Introduction of free dental check-ups to patients aged <25 y and >60 y in 2001
- Introduction of a new NHS dental contract in 2006 with loss of patient registration and capitation payments and introduction of a new payment model for NHS dentists. In the same year, there was also change in provision of dental care in Wales, with responsibility for providing care moved to local health boards from individual practices.
- Introduction of NHS Direct Wales in 2007, a free-to-use national telephone service for advice and access to nonemergency medical and dental services.

The aims of this study were to describe 44-y trends in dental GMP attendances in Wales, specifically in relation to dental policy change, and to identify factors associated with repeat attendance during the period.

## Materials and Methods

A retrospective observational study was completed with the general practitioner data set within the Secure Anonymised Information Linkage (SAIL) Databank (Ford et al. 2009; Lyons et al. 2009). SAIL is a national data set consisting of anonymized health and administrative data sets from the Welsh population, and it contains a “general practitioner data set” beginning in 1974 with >40 y of data on Welsh GMP attendances. This gave annual cross-sectional data on patient attendances for each of the 44 y requested by the study team up to 2017. Approval was granted by the Health Information Research Unit’s Information Governance Review Panel.

Data were identified and extracted by a SAIL analyst. At the time of data extraction, the data set covered 76.9% of GMP practices. All patient attendances for dental problems were included between January 1, 1974, and December 31, 2017. Identification of relevant patient attendances was based on dental and orofacial Read codes (version 2; Appendix Table 1). Read codes are a clinical terminology used in general medical practice in the United Kingdom and are based on medical terms. They include and cross-reference all of the other widely used medical classifications, and they are used to code details of multiple demographics, investigations, therapeutics, and operative treatments of individual patients (Chisholm 1990). Read codes for chronic orofacial pain were excluded.

For each Read code identified, the following covariates were extracted: patient ID, week of birth, gender, Welsh Index of Multiple Deprivation (WIMD) quintile, urban/rural classification, attendance date, and practice code. The WIMD is the official measure of relative deprivation in areas of Wales (Welsh Government 2011), and the 2001 urban/rural classification of the Office for National Statistics (2004) divides areas into urban and rural categories with subdivisions by sparsity (details available in the Appendix). The WIMD quintile and urban/rural classification for each patient attendance were provided by the SAIL team. Patient age was calculated by using week of birth and attendance date, and practice codes were used to calculate the number of dental patients per practice. For each dental attendance identified, associated antibiotic and referral Read codes were included to consider the impact of appointment outcome on whether the patient reattended. A complete list of referral and antibiotic Read codes is given in Appendix Table 1.

Rate of dental attendance was calculated as the number of attendances over time and converted into attendance rates per 1,000 patient-years per the Welsh Demographic Service data set (methodological details available in the Appendix). Denominator data were not available on patient location or age through this data set; as such, rates were not calculated separately for subgroups of age or location, and instead number of attendances was used.

Repeat attendance was defined as >1 attendance by the same patient in a 12-mo period, regardless of the practice attended. This definition was based on the assumption that if the first attendance was inappropriate, the GMP should have advised the patient to seek dental care, and subsequent GMP attendances that year would have been inappropriate.

Data cleaning was undertaken prior to analysis with Stata 15 (StataCorp LP) within the SAIL portal. To protect patients’ confidentiality, counts <5 were not exported from the portal; therefore, Read codes were grouped into larger diagnostic groups (Appendix Table 1). Where regrouping was not possible, counts were denoted as <5, and the total number for that variable was adjusted. Read codes relating to nonspecific dental diagnoses were grouped to form a “nonspecific dental diagnosis.” To examine predictors of repeat attendance, univariate and multivariable logistic regression modeling was performed. The binary response variable was whether a patient was a repeat attender by 12-mo period. Explanatory variables were gender, age, WIMD, urban/rural classification, antibiotic prescription, referral, and potential confounders, and interactions among age, gender, WIMD, and urban/rural were assessed. Statistical significance was determined through likelihood ratio tests. Stratified results are presented where a significant interaction was present (for each level of the interaction variable). For age, analysis was completed with categorical age groups within the regression model, and a fractional polynomial transformation was used for the continuous form. Regression modeling was repeated with adjustments for any potential confounders, which were included in the final model where a >10% change was observed.

## Results

Over the period studied, there were 439,361 Read codes associated with dental attendances at GMPs in Wales, accounting for 288,147 patient attendances, or 204,025 patients. The overall attendance rate was 2.60 attendances per 1,000 patient-years (95% CI, 2.59 to 2.61). The majority of patients attended once (39.61%) or twice (39.25%) during the 44-y period. The most common diagnoses were dental abscess (45.69%), nonspecific dental Read codes (29.18%), and toothache (14.70%). Patients tended to be from urban parts of Wales (59.55%), with overrepresentation of low-quintile WIMD (24.29%, WIMD 1) as compared with high-quintile WIMD (13.91%, WIMD 5). Detailed patient demographics are given in Appendix Tables 2 and 3.

**Figure 2.**
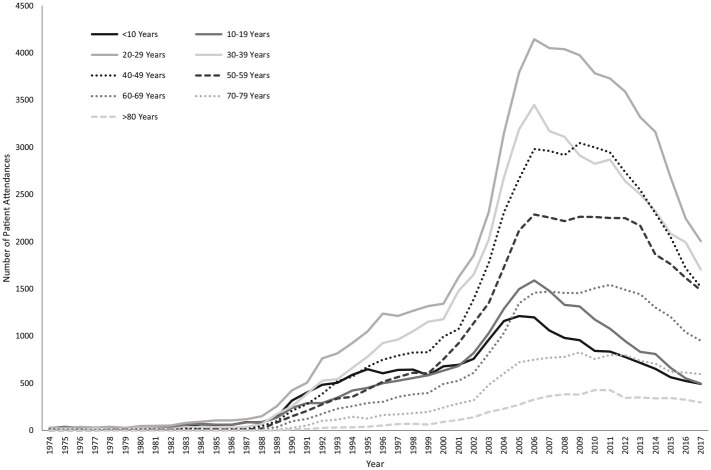
Ten-year age groups over the period studied. Solid lines indicate patient groups ≤39 y, and broken lines indicate patient groups ≥40 y.

**Figure 3.**
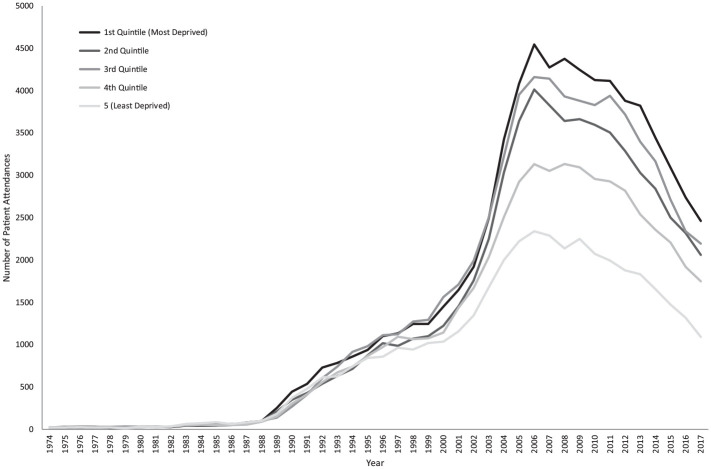
Changes in Welsh Index of Multiple Deprivation over the period studied.

The attendance rate over the 44-y period is shown in Figure 1 (the associated 95% CIs are shown in Appendix Fig. 1). At the peak of attendance in 2006, the rate was 5.01 attendances per 1,000 patient-years (95% CI, 4.94 to 5.09). The sociodemographic distribution of patients also changed, with mean patient age increasing from 20.80 to 42.09 y. In terms of age categories, attendances from all age groups increased initially, although more markedly in those between 20 and 49 y. From 2006, when overall attendance rates begin to decrease, the number of patients aged <39 y decreased immediately, whereas the number of patients ≥40 y decreased at a delayed rate (Fig. 2). Change in WIMD showed an increase across all WIMDs initially but more markedly from the lower quintiles, as well as a decrease from 2006 but with the lower quintiles still predominating (Fig. 3).

There were 37,985 repeat attendances representing 26,312 patients (12.90%). Repeat attendances were most commonly classified with a diagnosis of dental abscess (59.20%) or for an antibiotic prescription (38.90%). Repeat attenders resided in the most deprived and urban areas (Table 1).

**Table 1.** WIMD and Urban/Rural Classification of Repeat Attenders.

	No.	%
WIMD quintile		
1 (most deprived)	6,442	24.48
2	5,504	20.92
3	6,170	23.45
4	4,804	18.26
5 (least deprived)	3,392	12.89
Urban/rural classification		
Urban, less sparse	13,367	50.80
Urban, sparse	1,292	4.91
Town and fringe, less sparse	3,920	14.90
Town and fringe, sparse	2,068	7.86
Village, hamlet, and isolated dwellings; sparse	3,655	13.89
Village, hamlet, and isolated dwellings; less sparse	2,010	7.64

WIMD, Welsh Index of Multiple Deprivation.

Results of the full regression analysis is given in Appendix Table 4. Repeat attendance was associated with living in the most deprived areas: relative to the most deprived quintile, the odds ratio (OR) for repeat attendance was 0.87 in the least deprived quintile (95% CI, 0.86 to 0.89). Repeat attenders were also less likely to be from urban locations (OR, 0.84; 95% CI, 0.83 to 0.85; *P* < 0.0001). Within multivariable logistic regression, an interaction was noted between WIMD and rurality (LR test: LR χ^2^[4] = 376.64, *P* < 0.0001); the stratified analysis is shown in Table 2.

**Table 2.** Stratified Analysis for WIMD and Urban/Rural Classification for Repeat Attendance.

WIMD	Odds Ratio	95% CI
Urban^a^		
1	1.22	1.19 to 1.25
2	1.20	1.67 to 1.23
3	1.14	1.11 to 1.18
4	1.06	1.03 to 1.09
5	1.00 (Reference)	—
Rural^a^		
1	1.08	1.04 to 1.13
2	1.15	1.12 to 1.19
3	1.22	1.19 to 1.26
4	1.17	1.13 to 1.21
5	1.00 (Reference)	—

WIMD, Welsh Index of Multiple Deprivation.

a For each odds ratio (Nos. 1 to 4): *P* < 0.0001.

Increasing age up to 50 to 59 y was associated with increasing odds of being a repeat attender (Appendix Table 4). After this, increasing age had decreasing odds of being a repeat attender. Gender was not predictive of being a repeat attender (OR, 0.99; 95% CI, 0.98 to 1.00; *P* = 0.20).

Univariate analysis showed that prescription of an antibiotic on a first or subsequent visit increased the odds of being a repeat attender >2-fold (OR, 2.53; 95% CI, 2.50 to 2.56; *P* < 0.0001), with 27.62% of initial appointments and 48.55% of repeat attendances including a Read code for an antibiotic prescription. Referral to another service decreased the odds of being a repeat attender (OR, 0.75; 95% CI, 0.70 to 0.81; *P* < 0.0001), with 0.80% of initial appointments and 0.50% of repeat attendances with a referral Read code. There was no evidence of confounding within the multivariable regression modeling (Appendix Table 4).

## Discussion

Over the period studied, there were large increases in the numbers of patients, followed by large decreases that coincided with changes in dental policy and program delivery (Fig. 1). These changes in attendance patterns were more pronounced in certain patient groups, such as those <40 y old and those from the most deprived areas of Wales. A total of 12.90% of patients were repeat attenders, and predictors for this included living in rural locations or deprived and urban areas. Appointment outcome was predictive of repeat attendance, with an antibiotic prescription increasing repeat attendance and a referral decreasing attendance.

Limitations to this study include findings heavily relying on accurate Read code reporting, as different GMPs and practices may have different coding habits. Indeed, standard rules for recording clinical codes in primary care do not exist (SAIL Databank 2021). At the start of the study period, there may have been lower numbers of practices with computerized patient management systems, and data for some patients may be underrecorded. This has, however, been taken into account by use of attendance rates. Given the large number of nonspecific dental Read codes, it is possible that incorrect diagnostic Read codes could have been used for dental patients. These nonspecific dental Read codes were, however, still labeled dental diagnoses; therefore, the overall rates presented are likely to be accurate. There may, however, be bias within the breakdown of specific dental diagnoses, therefore we did not carry out detailed analysis of individual diagnostic groups. Other important predictors or confounders, such as the oral health status of the patient, were not available to include in the data set for analysis. This may have introduced bias, and additional research with these indicators would be warranted. Use of WIMD for deprivation levels in rural Wales has been criticized, as deprived people in these areas tend to be more geographically dispersed and may be disproportionately affected by some of the deprivation indicators used in comparison with those living in urban areas (Jones 2015). This could mean that deprivation is still an indicator for repeat attendance in rural areas but is not reflected in the model due to the use of WIMD. Finally, the changes seen in attendance rates over the period studied may have been caused by policy change, but investigating this further was not possible due to there being multiple policy changes, as well as potential delays in their effect and geographic variation within Wales. Again this is an area that warrants more research. A strength of this study is the large sample size that was available for analysis over a long period, meaning that issues with statistical power were not a concern and external validity of the study is increased.

Dental policy changes in Wales and the rest of the United Kingdom may partly explain some of the changes observed over time (Fig. 1). In 1990 changes were made to UK dental services (National Health Service 1990) resulting in an increase in GDP patient registration (Tickle 2012), which is unlikely to explain the increase in attendances from 1990. However, additional contractual changes were made in 1992 leading to problems with dental access peaking by 2004 (Tickle 2012). This could explain the increase from 1992. In 2001 Welsh policy introduced free dental check-ups for patients aged <25 and >60 y (National Health Service 2006); this appears not to have had an effect on dental GMP attendances, unless it had been masked by dental access issues. In 2006, there was a UK-wide NHS dental policy change (National Health Service 2005), largely reported as encouraging GDPs to move from NHS to private dental practice (Tickle 2012). This could have worsened access problems, but this does not coincide with the attendance rates observed, as from 2006 GMP dental attendances started to decline. It may, however, partly explain some of the increased attendances pre-2006 when GDPs became aware of the planned contract change and began to move into the private sector, but it is unlikely to fully explain the increased rates observed. In 2006 in Wales, there was a change in responsibility for determining where new dental practices would be set up and for urgent dental care provision (National Assembly for Wales 2016). As a result, dental access was improved in more deprived areas, and an increase in patients accessing urgent dental care was seen (StatsWales 2019), which may explain the reduced rate of attendances to GMPs. In 2007 NHS Direct Wales was introduced as a free national telephone service for nonemergency health care, but only some local health boards adopted this for urgent dental care (Cope 2016). This could partly explain the reduction in attendances, particularly in the younger patients via an improvement in health literacy, as most patients accessing this service are from younger age groups (Welsh Ambulance Service NHS Trust 2019).

Change in attendance patterns was more pronounced in certain patient groups. The decrease in attendance rates from 2006 was initially observed in those <40 y old. Attendances in patients aged >70 y appeared to remain stable with no obvious decrease noted; therefore, this elderly group either could not change their attendance behavior or were not willing to. Older adults are more likely to have comorbidities for which they would be regularly seeing their GMP and, as such, are more likely to have established long-term doctor-patient relationships, which may account for this. A sharper increase in attendances from those in deprived areas was noted per the WIMD, which may indicate an increase in oral health inequalities that did not fully resolve by 2017. The decrease from 2006 appears to affect the more deprived quintiles, with a more gradual decrease in attendances noted from those in the less deprived areas. This may indicate that whatever initiated the change in attendance rate from 2006 had the biggest impact on younger adults living in deprived areas.

Reasons why patients may seek dental care from a GMP rather than a GDP are multifactorial. Patient self-reported reasons include: understanding of symptoms, perceptions of the scope of practice between the GMP and GDP, complexities and unfamiliarity with dental care systems, availability of (urgent) dental care, dental anxiety, dissatisfaction with previous dental care, and willingness and ability to pay for dental treatment (Cohen, Harris, et al. 2009; Cope, Butt, and Chestnutt 2018; Cope, Wood, et al. 2018). GMP-reported views are as follows: dental access or comparative ease of medical access, practitioner preference, financial concerns, perceived need for antibiotics, and referred or poorly differentiated pain (Cope et al. 2015). In addition, GMPs have reported seeing increases in dental patients with disruption of local dental services and a decrease when dental access or practice triaging systems are improved or with health care signposting interventions (Cope et al. 2015). Although these reasons may explain why a patient would choose to seek care from a GMP for dental pain rather than a GDP, not all of them will explain the changes in attendance patterns observed. For example, dental anxiety, dissatisfaction with previous care, practitioner preference, and referred or poorly differentiated pain are all unlikely to change on a population level. In addition, although the ability to pay for dental treatment may change, willingness to pay may not. The remaining factors may cause population changes over time—for example, if a policy change or a population-level intervention is introduced.

Living in rural locations may make dental access challenging, where practices report difficulties in recruiting and retaining dentists (Owen et al. 2019). In addition, people living in rural areas are more likely to consider oral health as being less important (Heaton et al. 2004) and not attend a dentist regularly, but they are just as likely to seek care from their GMPs as their urban counterparts (Khan et al. 2017).

Almost one-quarter of patients were from the most deprived areas of Wales, and deprivation was a predictor of repeat attendance in urban areas. The reasons why deprivation is associated with repeat attendance may include prevalence of dental disease and pain (Steele et al. 2011), fewer seeking regular dental care (Morris et al. 2011), increased prevalence of dental anxiety (Nuttall et al. 2011), poorer health literacy (Sørensen et al. 2015), and poorer general health leading to more frequent GMP care seeking (Dixon Woods et al. 2005). Frequent GMP consultations for comorbidities will improve access and may lead to better doctor-patient relationships, making dental care seeking from a GMP more likely (Cope, Butt, and Chestnutt 2018). In addition, patients from deprived backgrounds are given very little information on dental problems from their GMP (Cohen, Harris, et al. 2009). They also have shorter consultations, are less likely to be referred (Scott et al. 1996), and are more likely to be given a prescription for an antibiotic (Koller et al. 2013; Shallcross et al. 2017). Patients from deprived areas are additionally more likely to seek dental care from medical emergency departments (Currie et al. 2017); therefore, nondental attendances are not exclusive to GMPs.

Patients who were not referred to another service were more likely to become repeat attenders. If patients are unable to access the dental care that they require, they may reattend their GMP when their symptoms return or worsen. Prescription of an antibiotic was associated with repeat attendance, which has been reported previously (Cope et al. 2016). This also encourages repeat attendance for conditions such as throat and ear infections where antibiotics would not routinely be indicated (Little et al. 1997; Williamson et al. 2006), and GMPs report this to encourage repeat dental attendance (Cope et al. 2015). However, in this data set, the majority of subsequent attendances were for prescription of antibiotics, which could indicate reverse causation, with patients reattending when symptoms do not resolve, resulting in an antibiotic prescription.

The decrease in dental GMP attendances from 2006 is not exclusive to Wales. Data analysis for the majority of England and small parts of the remainder of the United Kingdom revealed an increase in attendance rates between 2004 and 2008, followed by a decline to 2013 (Cope et al. 2016). This raises the possibility that the decrease noted in Wales is part of a UK-wide decrease, but long-term studies within the United Kingdom are required to establish this. Previous dental GMP attendance rates in Wales were reported as being higher at 6.90 per 1,000 patient-years in 1996 (Anderson et al. 1999). This can be explained by the size of the SAIL data set at the time of analysis and inclusion of all orofacial and chronic orofacial pain Read codes, such as diseases of the salivary glands and temporomandibular disorders. In 1996, the SAIL data set covered only 0.3% of GMP practices in Wales; however, for this study it covered almost 80%. This includes addition of retrospective data once a practice begins to submit data to SAIL.

In conclusion, policy change may have an impact on dental patient attendances at GMPs, and predictors of repeat attendance include living in urban and deprived or rural areas, as well as the attendance outcome.

## Author Contributions

C.C. Currie, M.S. Pearce, contributed to conception, design, data analysis, and interpretation, drafted and critically revised the manuscript; S.J. Stone, P. Brocklehurst, J. Durham, contributed to conception, design, and data interpretation, critically revised the manuscript; G. Slade, contributed to data interpretation, critically revised the manuscript. All authors gave final approval and agree to be accountable for all aspects of the work.

## Supplemental Material

sj-docx-1-jdr-10.1177_00220345211044108 – Supplemental material for Dental Attendances to General Medical Practitioners in Wales: A 44 Year-Analysis Click here for additional data file. Supplemental material, sj-docx-1-jdr-10.1177_00220345211044108 for Dental Attendances to General Medical Practitioners in Wales: A 44 Year-Analysis by C.C. Currie, S.J. Stone, P. Brocklehurst, G. Slade, J. Durham and M.S. Pearce in Journal of Dental Research

## References

[bibr1-00220345211044108] Anderson R Richmond S Thomas DW . 1999. Patient presentation at medical practices with dental problems: an analysis of the 1996 general practice morbidity database for Wales. Br Dent J. 186(6):297–300. 10230104 10.1038/sj.bdj.4800091

[bibr2-00220345211044108] Bell GW Smith GL Rodgers JM Flynn RW Malone CH . 2008. Patient choice of primary care practitioner for orofacial symptoms. Br Dent J. 204(12):669–673. 18587362 10.1038/sj.bdj.2008.523

[bibr3-00220345211044108] Chisholm J. 1990. The read clinical classification. Br Med J. 300(6732):1092–1092. 2344534 10.1136/bmj.300.6732.1092 PMC1662793

[bibr4-00220345211044108] Cohen LA Bonito AJ Akin DR Manski RJ Macek MD Edwards RR Cornelius LJ . 2009. Role of pharmacists in consulting with the underserved regarding toothache pain. J Am Pharm Assoc. 49(1):38–42. 10.1331/JAPhA.2009.07149 19196595

[bibr5-00220345211044108] Cohen LA Harris SL Bonito AJ Manski RJ Macek MD Edwards RR Khanna N Plowden KO . 2009. Low-income and minority patient satisfaction with visits to emergency departments and physician offices for dental problems. J Am Coll Dent. 76(3):23–31. PMC2819232 19928365

[bibr6-00220345211044108] Cope A. 2016. Review of urgent and emergency dental care in Wales [accessed 2021 Aug 18]. https://www.ambulance.wales.nhs.uk/assets/documents/e5e029f8-5df2-49a6-a87e-3d765beb2db4636458390559076134.pdf

[bibr7-00220345211044108] Cope AL Butt KG Chestnutt IG . 2018. Why might patients in the UK consult a general medical practitioner when experiencing dental problems? A literature review of patients’ perspectives. Community Dent Health. 35(4):235–240. 30188615 10.1922/CDH_4369Cope06

[bibr8-00220345211044108] Cope AL Chestnutt IG Wood F Francis NA . 2016. Dental consultations in UK general practice and antibiotic prescribing rates: a retrospective cohort study. Br J Gen Pract. 66(646):e329–e336. 27025554 10.3399/bjgp16X684757 PMC4838445

[bibr9-00220345211044108] Cope AL Wood F Francis NA Chestnutt IG . 2015. General practitioners’ attitudes towards the management of dental conditions and use of antibiotics in these consultations: a qualitative study. BMJ Open. 5(10):e008551. 10.1136/bmjopen-2015-008551 PMC4606392 26428331

[bibr10-00220345211044108] Cope AL Wood F Francis NA Chestnutt IG . 2018. Patients’ reasons for consulting a GP when experiencing a dental problem: a qualitative study. Br J Gen Pract. 68(677):e877–e883. 30348888 10.3399/bjgp18X699749 PMC6255242

[bibr11-00220345211044108] Currie CC Stone SJ Connolly J Durham J. 2017. Dental pain in the medical emergency department: a cross-sectional study. J Oral Rehabil. 44(2):105–111. 27896841 10.1111/joor.12462

[bibr12-00220345211044108] Dixon Woods M Kirk D Agarwal S Annadale E Arthur T Harvey J Hsu R Katbamma S Olsen R Smith L , et al. 2005. Vulnerable groups and access to health care: a critical interpretive review [accessed 2021 Aug 18]. https://citeseerx.ist.psu.edu/viewdoc/download?doi=10.1.1.126.8978&rep=rep1&type=pdf

[bibr13-00220345211044108] Ford DV Jones KH Verplancke JP Lyons RA John G Brown G Brooks CJ Thompson S Bodger O Couch T , et al. 2009. The SAIL Databank: building a national architecture for e-health research and evaluation. BMC Health Serv Res. 9(1):157. 19732426 10.1186/1472-6963-9-157 PMC2744675

[bibr14-00220345211044108] General Practitioners Committee. 2007. Patients presenting with dental problems: GP responsibilities [accessed 2021 Aug 18]. https://www.gloslmc.com/downloads/Dental/Patients%20presenting%20with%20dental%20problems%2007.pdf

[bibr15-00220345211044108] Heaton LJ Smith TA Raybould TP . 2004. Factors influencing use of dental services in rural and urban communities: considerations for practitioners in underserved areas. J Dent Educ. 68(10):1081–1089. 15466058

[bibr16-00220345211044108] Jones L. 2015. Welsh index of multiple deprivation 2014: a guide to analysing deprivation in rural areas [accessed 2021 Aug 18]. https://gov.wales/sites/default/files/statistics-and-research/2019-05/welsh-index-of-multiple-deprivation-2014-a-guide-to-analysing-deprivation-in-rural-areas.pdf

[bibr17-00220345211044108] Khan A Thapa JR Zhang D. 2017. Preventive dental checkups and their association with access to usual source of care among rural and urban adult residents. J Rural Health. 33(4):419–426. 28905468 10.1111/jrh.12271

[bibr18-00220345211044108] Koller D Hoffmann F Maier W Tholen K Windt R Glaeske G. 2013. Variation in antibiotic prescriptions: is area deprivation an explanation? Analysis of 1.2 million children in Germany. Infection. 41(1):121–127. 22826031 10.1007/s15010-012-0302-1

[bibr19-00220345211044108] Little P Gould C Williamson I Warner G Gantley M Kinmonth AL . 1997. Reattendance and complications in a randomised trial of prescribing strategies for sore throat: the medicalising effect of prescribing antibiotics. Br Med J. 315(7104):350–352. 9270458 10.1136/bmj.315.7104.350 PMC2127265

[bibr20-00220345211044108] Lyons RA Jones KH John G Brooks CJ Verplancke JP Ford DV Brown G Leake K. 2009. The SAIL Databank: linking multiple health and social care datasets. BMC Med Inform Decis Mak. 9:3. 19149883 10.1186/1472-6947-9-3 PMC2648953

[bibr21-00220345211044108] Morris J Chenery J Douglas G Treasure E. 2011. Service considerations – a report from the Adult Dental Health survey 2009 [accessed 2021 Aug 18]. https://files.digital.nhs.uk/publicationimport/pub01xxx/pub01086/adul-dent-heal-surv-summ-them-the6-2009-rep8.pdf

[bibr22-00220345211044108] National Assembly for Wales. 2016. New publication: dentistry in Wales [accessed 2021 Aug 18]. https://seneddresearch.blog/2016/06/28/new-publication-dentistry-in-wales/

[bibr23-00220345211044108] National Health Service. 1990. National Health Service (general dental Services) (miscellaneous amendments) regulations [accessed 2021 Aug 18]. https://www.legislation.gov.uk/uksi/1990/1638/contents/made

[bibr24-00220345211044108] National Health Service. 2005. National Health Service (dental charges) regulations 2005 [accessed 2021 Aug 18]. https://www.legislation.gov.uk/uksi/2005/3477/made

[bibr25-00220345211044108] National Health Service. 2006. National Health Service (dental charges) (Wales) regulations [accessed 2021 Aug 18]. https://www.legislation.gov.uk/wsi/2006/491/contents/made

[bibr26-00220345211044108] Nuttall N Freeman R Beavan-Seymour C Hill K. 2011. Access and barriers to care – a report from the Adult Dental Health Survey 2009 [accessed 2021 Aug 18]. https://files.digital.nhs.uk/publicationimport/pub01xxx/pub01086/adul-dent-heal-surv-summ-them-the8-2009-re10.pdf

[bibr27-00220345211044108] Office for National Statistics. 2004. 2001 Rural-urban classification – Office for National Statistics [accessed 2021 Aug 18]. https://cy.ons.gov.uk/methodology/geography/geographicalproducts/ruralurbanclassifications/2001ruralurbanclassification

[bibr28-00220345211044108] Owen C Seddon C Clarke K Bysouth T. 2019. NHS general dentistry in Wales: evaluation of patient access and budget expenditure. Br Dent J. 226(12):967–978. 31253919 10.1038/s41415-019-0407-3

[bibr29-00220345211044108] Reda SM Krois J Reda SF Thomson WM Schwendicke F. 2018. The impact of demographic, health-related and social factors on dental services utilization: systematic review and meta-analysis. J Dent. 75:1–6. 29673686 10.1016/j.jdent.2018.04.010

[bibr30-00220345211044108] SAIL Databank. 2021. SAIL Databank – primary care GP dataset [accessed 2021 Aug 18]. https://saildatabank.com/saildata/sail-datasets/primary-care-gp-dataset/

[bibr31-00220345211044108] Scott A Shiell A King M. 1996. Is general practitioner decision making associated with patient socio-economic status? Soc Sci Med. 42(1):35–46. 8745106 10.1016/0277-9536(95)00063-1

[bibr32-00220345211044108] Shallcross L Beckley N Rait G Hayward A Petersen I. 2017. Antibiotic prescribing frequency amongst patients in primary care: a cohort study using electronic health records. J Antimicrob Chemother. 72(6):1818–1824. 28333200 10.1093/jac/dkx048 PMC5437523

[bibr33-00220345211044108] Sørensen K Pelikan JM Röthlin F Ganahl K Slonska Z Doyle G Fullam J Kondilis B Agrafiotis D Uiters E , et al. 2015. Health literacy in Europe: comparative results of the European health literacy survey (HLS-EU). Eur J Public Health. 25(6):1053–1058. 25843827 10.1093/eurpub/ckv043 PMC4668324

[bibr34-00220345211044108] StatsWales. 2019. Courses of treatment and units of dental activity (UDA) by local health board and treatment band [accessed 2021 Aug 18]. https://statswales.gov.wales/Catalogue/Health-and-Social-Care/General-Dental-Services/Current-Contract/Pre-October-2009/CoursesOfTreatmentAndUnitsOfDentalActivity-by-LocalAuthority-TreatmentBand

[bibr35-00220345211044108] Steele JG Pitts N Fuller E Treasure E. 2011. Urgent conditions – a report from the Adult Dental health survey 2009 [accessed 2021 Aug 18]. https://files.digital.nhs.uk/publicationimport/pub01xxx/pub01086/adul-dent-heal-surv-summ-them-the3-2009-rep5.pdf

[bibr36-00220345211044108] Tickle M. 2012. Revolution in the provision of dental services in the UK. Community Dent Oral Epidemiol. 40 Suppl 2:110–116. 10.1111/j.1600-0528.2012.00729.x 22998314

[bibr37-00220345211044108] Welsh Ambulance Service NHS Trust. 2019. Freedom of information request. Reference 17819.

[bibr38-00220345211044108] Welsh Government. 2011. Welsh index of multiple deprivation 2011: guidance on use [accessed 2021 Aug 18]. https://gov.wales/sites/default/files/statistics-and-research/2019-04/wimd-2011-guidance-on-use.pdf

[bibr39-00220345211044108] Williamson I Benge S Mullee M Little P. 2006. Consultations for middle ear disease, antibiotic prescribing and risk factors for reattendance: a case-linked cohort study. Br J Gen Pract. 56(524):170–175. 16536956 PMC1828259

