# Supplementary material for: Dental Attendances to General Medical Practitioners in Wales: A 44 Year-Analysis
Source: J Dent Res. 2021 Sep 28;101(4):407–13. doi: 10.1177/00220345211044108 (PMC8935529; doi:10.1177/00220345211044108)
Supplement: sj-docx-1-jdr-10.1177_00220345211044108 – Supplemental material for Dental Attendances to General Medical Practitioners in Wales: A 44 Year-Analysis [file sj-docx-1-jdr-10.1177_00220345211044108.docx]

**Dental Attendances to General Medical Practitioners in Wales: a 44 Year Analysis**

Currie CC, Stone SJ, Brocklehurst P, Slade G, Durham J, Pearce M

**Supplemental Methodological Details**

**Welsh Index of Multiple Deprivation (WIMD)**

WIMD is the official measurement of deprivation of areas of Wales (Welsh Government, 2011) and takes into account eight different domains of deprivation: employment; income; education; health; community safety; geographical access to services; housing and physical environment. WIMD quintiles were used for analysis with quintile 1 being the 20% most deprived areas, and quintile 5 being the 20% least deprived areas of Wales. The SAIL team provided the WIMD quintile for each patient using lower super output area (LSOA) mapped to the same 2011 WIMD measure to allow comparison of relative deprivation over time.

**Office for National Statistics Urban/Rural Classification 2001**

The Office for National Statistics Urban/Rural classification 2001 (Office for National Statistics, 2016) divides geographical areas in urban (physical settlements with a population more than 10,000) and rural categories, with further subdivisions by settlement type and sparsity. Settlements in sparse areas have a particularly low number of households (compared to those defined as less sparse) and therefore may have implications on service availability.

**Calculation of Attendance Rates**

The attendance rate was calculated as the annual rate of dental consultations per 1000 patient-years to allow direct comparison with previous studies. This is similar to calculating an incidence rate. The denominator to calculate the attendance rates was calculated using data from the Welsh Demographic Service (WDS) dataset available through SAIL. This patient level dataset contains all primary care events coded with Read codes by GMPs. This allowed calculation of the total patient-years for all attendances at all GMPs registered with SAIL by year. For partial annual data, e.g. where a patient entered the dataset part way through a calendar year, the amount of time they had contributed to that year was included by calculation of the proportion. For example, where a patient had entered the dataset in the 7th month of the year they were considered to have contributed 0.5 patient-years (6 months). Data were not available from the WDS dataset on patient location or age, therefore incidence rates could not be calculated for these variables.

**Supplemental Appendix Tables**

**Appendix Table 1:** Read codes used for data extraction and re-grouping of Read codes. Note: The Read code for clindamycin was included in the search however there were no observations remaining for this following data cleaning.

| Read Code | Read Code Description | Group |
| --- | --- | --- |
| 1912 | Toothache | Toothache |
| J020 | Pulpitis |  |
| J0200 | Pulpal abscess |  |
| J024 | Acute apical periodontitis |  |
| 1914 | Dental swelling | Dental abscess |
| J0250 | Dental abscess |  |
| J0251 | Dentoalveolar abscess |  |
| J0332 | Paradental abscess |  |
| J083 | Oral cellulitis and abscess |  |
| 75112 | Surgical removal of wisdom tooth | Pericoronitis |
| J0331 | Acute pericoronitis |  |
| J0340 | Chronic pericoronitis |  |
| 1913 | Bad teeth/caries | Caries |
| J010 | Dental caries |  |
| J01y1 | Sensitive tooth dentine | Dentine Hypersensitivity |
| J03.. | Gingival/periodontal disease | Gingival/Periodontal diseases |
| J065 | Alveolitis of jaw | Alveolitis of jaw |
| J080 | Stomatitis | Stomatitis |
| S8363 | Broken tooth injury | Dental trauma |
| 191.. | Tooth symptoms | Non-specific dental Read codes |
| J05y | Other specified dental disorder |  |
| J052 | Dental diseases/conditions |  |
| 8HT4 | Referral to orthodontic clinic | Referral to orthodontics |
| 8HoA | Referral orthodontic service |  |
| 8Hv9 | Private referral to oral surgeon | Private referral to oral/maxillofacial surgeon |
| 8HVD | Private referral to maxfax |  |
| 8Hn13 | Fast track referral head and neck cancer | Fast track referral head and neck cancer |
| 8Ho4 | Referral oral surgery service | Referral to oral surgeon |
| 8Ho1 | Referral restorative dental service | Referral to specific dental service |
| 8Ho2 | Referral dental conservation service |  |
| 8Ho3 | Referral paediatric dental service |  |
| H8o5 | Referral endodontic service |  |
| 8Ho6 | Referral dental sedation service |  |
| 8Ho7 | Referral periodontal service |  |
| 8Ho8 | Referral prosthodontic service |  |
| 8Ho9 | Referral dental radiology service |  |
| 8Hd | Admission to hospital | Admission to hospital |
| 8H2k.. | Admission oral surgery emergency | Admission oral surgery emergency |
| 8H3u | Non urgent oral surgery admission | Non urgent oral surgery admission |
| 8HT | Referral to clinic | Non-specific referral |
| 8HTE | Referral to other clinic |  |
| 8HTZ | Referral to clinic NOS |  |
| 8H2 | Further care referral NOS |  |
| 8H20 | Referral needed |  |
| 8Hk | Referred to service |  |
| 8Hkj | Informal referral, signposted to other agency |  |
| 8H1 | Referral to other care |  |
| 8IH5.. | Referral declined | Referral declined |
| e15.. | Phenoxymethylpenicillin | Phenoxymethylpenicillin |
| e3.. | Broad spectrum penicillin | Broad spectrum penicillin |
| e3z.. & e3a.. | Amoxicillin | Amoxicillin |
| e91.. & e95.. | Erythromycin | Erythromycin |
| e1.. | Metronidazole | Metronidazole |
| 8BGB.. | Antibiotic indicated | Antibiotic indicated |

**Appendix Table 2:** Detailed patient demographics from the entire dataset (1974-2017).

| **Patient Gender, n (%)** | |
| --- | --- |
| Male | 130,139 (45.16%; 2.37 attendances per 1000 patient-years) |
| Female | 158,008 (54.84%; 2.85 attendances per 1000 patient-years) |
| Indeterminate/anticipated sex change | 0 |
| Not known | 0 |
| **Patient Age** | |
| Mean age (years) | 38.32 (SD 19.21) |
| Age range (years) | 0 - >100 (exact age range not permitted due to counts <5) |
| **WIMD Quintile, n (%)** | |
| 1 (most deprived) | 69,995 (24.29) |
| 2 | 60,139 (20.87) |
| 3 | 65,879 (22.86) |
| 4 | 52,048 (18.06) |
| 5 (least deprived) | 40,086 (13.91) |
| **Urban/Rural Definition, n (%)** | |
| Urban; sparse | 13,968 (4.85) |
| Urban; less sparse | 157,634 (54.71) |
| Town & fringe; sparse | 18,183 (6.31) |
| Town & fringe; less sparse | 42,417 (14.72) |
| Village, hamlet & isolated dwellings; sparse | 34,848 (12.09) |
| Village, hamlet & isolated dwellings; less sparse | 21,097 (7.32) |

**Appendix Table 3**: Detailed patient demographics by diagnosis over the entire dataset (1974-2017). **Note: counts <5 were present in subgroup analysis therefore subtotals vary across demographic variables.**

|  | **Dental abscess** | **Toothache** | **Caries** | **Pericoronitis** | **Stomatitis** | **Alveolitis of jaw** | **Gingival/Periodontal Disease** | **Dental trauma** | **Non-specific dental Read codes** | **All diagnoses** |
| --- | --- | --- | --- | --- | --- | --- | --- | --- | --- | --- |
| **Gender, n (%)** | | | | | | | | | |  |
| Male | 60,058 (47.72) | 17,728 (43.78) | 5,636 (53.81) | 2,556 (33.61) | 2,542 (39.07) | 785 (43.93) | 728 (43.36) | 214 (33.33) | 34,933 (43.46) | 125,370 (45.52) |
| Female | 65,797 (52.28) | 22,766 (56.22) | 4,837 (46.19) | 5,050 (66.39) | 3,965 (60.93) | 1,002 (56.07) | 951 (56.64) | 428 (66.67) | 45,447 (56.54) | 150,045 (54.48) |
| **WIMD, n (%)** | | | | | | | | | |  |
| 1 | 31.941 (25.38) | 9,385 (23,18) | 3,618 (34.55) | 1,165 (15.32) | 1,385 (21.28) | 540 (30.22) | 368 (21.92) | 161 (25.08) | 18,427 (22.92) | 67,002 (24.32) |
| 2 | 26,848 (21.33) | 8,864 (21.89) | 2,413 (23.04) | 1,370 (18.01) | 1,179 (18.12) | 498 (27.87) | 302 (17.99) | 151 (23.52) | 15,885 (19.76) | 57,520 (20.88) |
| 3 | 28,936 (22.99) | 9,650 (23.83) | 2,190 (20.91) | 1,562 (20.54) | 1,448 (22.25) | 333 (18.63) | 391 (23.29) | 133 (20.72) | 18,177 (22.61) | 62,833 (22.81) |
| 4 | 22,354 (17.76) | 7,043 (17.39) | 1,412 (13.48) | 1,450 (19.06) | 1,168 (17.95) | 215 (12.03) | 311 (18.52) | 97 (15.11) | 15,739 (19.58) | 49,798 (18.08) |
| 5 | 15,776 (12.54) | 5,552 (13.71) | 840 (8.02) | 2,059 (27.07) | 1,327 (20.39) | 201 (11.25) | 307 (18.28) | 642 (15.58) | 12,152 (15.12) | 38,319 (13.91) |
| **Age Group, years n (%)** | | | | | | | | | |  |
| <10 | 7,639 (6.07) | 2,217 (5.47) | 2,689 (25.68) | 32 (0.42) | 1,895 (29.12) | 101 (5.65) | 102 (6.08) | 205 (31.93) | 5,430 (6.76) | 20,310 (7.37) |
| 10-19 | 9,530 (7.57) | 3,266 (8.07) | 918 (8.77) | 1,244 (16.36) | 621 (9.54) | 143 (8.00) | 138 (8.22) | 148 (23.05) | 6,430 (8.00) | 22,438 (8.15) |
| 20-29 | 27,471 (21.83) | 10,430 (25.76) | 1,715 (16.38) | 4,216 (55.43) | 576 (8.85) | 377 (21.10) | 301 (17.93) | 92 (14.33) | 18,182 (22.62) | 63,360 (23.00) |
| 30-39 | 23,976 (19.05) | 7,693 (19.00) | 1,494 (14.27) | 1,389 (18.26) | 541 (8.31) | 329 (18.41) | 262 (15.60) | 75 (11.68) | 13,735 (17.09) | 49,494 (17.97) |
| 40-49 | 23,306 (18.52) | 6,451 (15.93) | 1,252 (11.95) | 443 (5.82) | 537 (8.25) | 277 (15.50) | 275 (16.38) | 30 (4.67) | 12,340 (15.35) | 44,911 (16.31) |
| 50-59 | 17,760 (14.11) | 4,832 (11.93) | 952 (9.09) | 173 (2.27) | 576 (8.85) | 265 (14.83) | 248 (14.77) | 31 (4.83) | 10,289 (12.80) | 35,126 (12.75) |
| 60-69 | 10,068 (8.00) | 3,098 (7.65) | 707 (6.75) | 71 (0.93) | 757 (11.63) | 161 (9.01) | 201 (11.97) | 17 (2.65) | 7,364 (9.16) | 22,444 (8.15) |
| 70-79 | 4,500 (3.58) | 1,746 (4.31) | 457 (4.36) | 30 (0.39) | 605 (9.30) | 105 (5.88) | 100 (5.96) | 29 (4.51) | 4,418 (5.50) | 11,990 (4.35) |
| >80 | 1,605 (1.28) | 761 (1.88) | 289 (2.76) | 8 (0.11) | 399 (6.13) | 29 (1.62) | 52 (3.09) | 15 (2.34) | 2,192 (2.73) | 5,350 (1.94) |
| **All patients**  **N (%)** | 125,855 (45.69) | 40,494 (14.70) | 10,473 (3.80) | 7,606 (2.76) | 6,507 (2.36) | 1,787 (0.65) | 1,679 (0.61) | 642 (0.23) | 80,380 (29.18) |  |

**Appendix Table 4:** Logistic regression to identify potential confounders for repeat attendances with adjustments in multivariable model.

| **WIMD** | **Univariate Analysis** | | | **Adjusted for Age** | | | **Adjusted for Urban/Rural** | | | **Adjusted for Gender** | | | **Adjusted for WIMD** | | |
| --- | --- | --- | --- | --- | --- | --- | --- | --- | --- | --- | --- | --- | --- | --- | --- |
|  | **OR** | **95% CI** | **P Value** | **OR** | **95% CI** | **P Value** | **OR** | **95% CI** | **P Value** | **OR** | **95% CI** | **P Value** | **OR** | **95% CI** | **P Value** |
| WIMD | | | | | | | | | | | | | | | |
| 1 | 1.00 (ref) |  |  |  |  |  |  |  |  |  |  |  |  |  |  |
| 2 | 1.02 | 1.01-1.04 | <0.05 | 1.01 | 1.00-1.03 | 0.112 | 0.99 | 0.97-1.01 | 0.239 | 1.02 | 1.01-1.04 | <0.05 |  |  |  |
| 3 | 1.08 | 1.06-1.10 | <0.0001 | 1.06 | 1.04-1.08 | <0.0001 | 0.99 | 0.97-1.01 | 0.372 | 1.08 | 1.06-1.10 | <0.0001 |  |  |  |
| 4 | 1.02 | 1.01-1.04 | <0.01 | 1.00 | 0.98-1.02 | 0.959 | 0.94 | 0.92-0.96 | <0.0001 | 1.03 | 1.01-1.04 | <0.05 |  |  |  |
| 5 | 0.87 | 0.86-0.89 | <0.0001 | 0.85 | 0.83-0.87 | <0.0001 | 0.83 | 0.82-0.85 | <0.0001 | 0.87 | 0.85-0.89 | <0.0001 |  |  |  |
| Urban/Rural | | | | | | | | | | | | | | | |
| Urban | 1.00 (ref) |  |  |  |  |  |  |  |  |  |  |  |  |  |  |
| Rural | 0.84 | 0.86-0.89 | <0.0001 | 0.85 | 0.84-0.86 | <0.0001 |  |  |  | 0.84 | 0.83-0.85 | <0.0001 | 0.83 | 0.82-0.84 | <0.0001 |
| Gender | | | | | | | | | | | | | | | |
| Male | 1.00 (ref) |  |  |  |  |  |  |  |  |  |  |  |  |  |  |
| Female | 0.99 | 0.98-1.00 | 0.20 | 1.00 | 0.98-1.01 | 0.422 | 0.99 | 0.98-1.00 | 0.402 |  |  |  | 0.99 | 0.98-1.00 | 0.154 |
| Age Group | | | | | | | | | | | | | | | |
| <10 | 1.00 (ref) |  |  |  |  |  |  |  |  |  |  |  |  |  |  |
| 10-19 | 1.31 | 1.26-1.35 | <0.0001 |  |  |  | 1.30 | 1.26-1.35 | <0.0001 | 1.31 | 1.26-1.35 | <0.0001 | 1.31 | 1.26-1.35 | <0.0001 |
| 20-29 | 1.56 | 1.52-1.61 | <0.0001 |  |  |  | 1.57 | 1.52-1.61 | <0.0001 | 1.56 | 1.52-1.61 | <0.0001 | 1.56 | 1.52-1.61 | <0.0001 |
| 30-39 | 1.70 | 1.65-1.75 | <0.0001 |  |  |  | 1.69 | 1.65-1.74 | <0.0001 | 1.70 | 1.65-1.75 | <0.0001 | 1.69 | 1.65-1.74 | <0.0001 |
| 40-49 | 1.76 | 1.70-1.80 | <0.0001 |  |  |  | 1.74 | 1.69-1.79 | <0.0001 | 1.76 | 1.71-1.81 | <0.0001 | 1.76 | 1.71-1.81 | <0.0001 |
| 50-59 | 1.75 | 1.70-1.80 | <0.0001 |  |  |  | 1.73 | 1.67-1.78 | <0.0001 | 1.75 | 1.70-1.80 | <0.0001 | 1.75 | 1.70-1.80 | <0.0001 |
| 60-69 | 1.67 | 1.62-1.73 | <0.0001 |  |  |  | 1.64 | 1.59-1.70 | <0.0001 | 1.67 | 1.62-1.73 | <0.0001 | 1.68 | 1.63-1.74 | <0.0001 |
| 70-79 | 1.49 | 1.43-1.54 | <0.0001 |  |  |  | 1.46 | 1.40-1.52 | <0.0001 | 1.49 | 1.43-1.54 | <0.0001 | 1.50 | 1.44-1.56 | <0.0001 |
| >80 | 1.31 | 1.25-1.38 | <0.0001 |  |  |  | 1.29 | 1.22-1.36 | <0.0001 | 1.31 | 1.25-1.38 | <0.0001 | 1.33 | 1.26-1.40 | <0.0001 |
| Antibiotic Prescription | | | | | | | | | | | | | | | |
| Yes | 1.00 (ref) |  |  |  |  |  |  |  |  |  |  |  |  |  |  |
| No | 2.53 | 2.50-2.56 | <0.0001 | 2.52 | 2.49-2.55 | <0.0001 | 2.52 | 2.49-2.55 | <0.0001 | 2.53 | 2.50-2.56 | <0.0001 | 2.53 | 2.49-2.56 | <0.0001 |
| Referral | | | | | | | | | | | | | | | |
| Yes | 1.00 (ref) |  |  |  |  |  |  |  |  |  |  |  |  |  |  |
| No | 0.75 | 0.70-0.81 | <0.0001 | 0.74 | 0.69-0.80 | <0.0001 | 0.78 | 0.72-0.84 | <0.0001 | 0.75 | 0.70-0.81 | <0.0001 | 0.76 | 0.70-0.82 | <0.0001 |

**Supplemental Appendix Figures**

**Appendix Figure 1:** Attendance rates over the time period with their associated 95% confidence intervals.


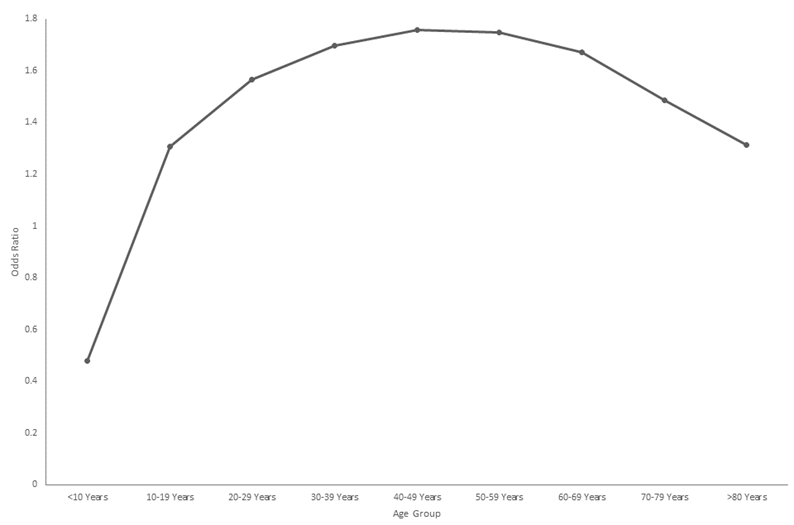


**Appendix Figure 2:** Odds ratio using the fractional polynomial transformation for being a repeat dental attender related to age group (all p<0.0001).

Fractional polynomial equation: y = 8292043+ (ln(X)+.946) + (X^3-.058), where x = (age+.002/100), where y is the odds of repeat attendance.
